# Histamine is a modulator of metamorphic competence in *Strongylocentrotus purpuratus* (Echinodermata: Echinoidea)

**DOI:** 10.1186/1471-213X-12-14

**Published:** 2012-04-27

**Authors:** Josh Sutherby, Jamie-Lee Giardini, Julia Nguyen, Gary Wessel, Mariana Leguia, Andreas Heyland

**Affiliations:** 1 University of Guelph, Integrative Biology, Guelph, ON N1G-2 W1, Canada; 2 Brown University, MCB, Providence, RI 02912, USA; 3 Current address: U.S. Naval Medical Research Unit No.6, Lima, Peru

**Keywords:** Metamorphosis, Metamorphic competence, Settlement, Echinoderm life-history, Histamine, Histamine receptors, Settlement, Life history evolution, Modulation

## Abstract

**Background:**

A metamorphic life-history is present in the majority of animal phyla. This developmental mode is particularly prominent among marine invertebrates with a bentho-planktonic life cycle, where a pelagic larval form transforms into a benthic adult. Metamorphic competence (the stage at which a larva is capable to undergo the metamorphic transformation and settlement) is an important adaptation both ecologically and physiologically. The competence period maintains the larval state until suitable settlement sites are encountered, at which point the larvae settle in response to settlement cues. The mechanistic basis for metamorphosis (the morphogenetic transition from a larva to a juvenile including settlement), i.e. the molecular and cellular processes underlying metamorphosis in marine invertebrate species, is poorly understood. Histamine (HA), a neurotransmitter used for various physiological and developmental functions among animals, has a critical role in sea urchin fertilization and in the induction of metamorphosis. Here we test the premise that HA functions as a developmental modulator of metamorphic competence in the sea urchin *Strongylocentrotus purpuratus*.

**Results:**

Our results provide strong evidence that HA leads to the acquisition of metamorphic competence in *S. purpuratus* larvae. Pharmacological analysis of several HA receptor antagonists and an inhibitor of HA synthesis indicates a function of HA in metamorphic competence as well as programmed cell death (PCD) during arm retraction. Furthermore we identified an extensive network of histaminergic neurons in pre-metamorphic and metamorphically competent larvae. Analysis of this network throughout larval development indicates that the maturation of specific neuronal clusters correlates with the acquisition of metamorphic competence. Moreover, histamine receptor antagonist treatment leads to the induction of caspase mediated apoptosis in competent larvae.

**Conclusions:**

We conclude that HA is a modulator of metamorphic competence in *S. purpuratus* development and hypothesize that HA may have played an important role in the evolution of settlement strategies in echinoids. Our findings provide novel insights into the evolution of HA signalling and its function in one of the most important and widespread life history transitions in the animal kingdom - metamorphosis.

## Background

Metamorphosis is a life history transition that is wide-spread in the animal kingdom and is abundantly present in marine invertebrate species [1,2]. Metamorphoses in these groups are often characterized by a rapid transition from a planktonic larval form to a benthic adult form (settlement) [3,4]. Directly preceding settlement is metamorphic competence (here also referred to as competence), a developmental state characterized by low metabolism and growth that can last for extensive periods of time (for extreme examples see [5-7]). Competence is followed by settlement, which in many cases is initiated by specific settlement cues from the environment. Note that pre-competent larvae generally do not respond to such settlement cues.

Echinoids (sea urchins and sand dollars) have evolved a broad range of life history strategies including significant variations in reproductive and developmental modes (reviewed in [2]). Understanding such evolutionary patterns requires detailed insights into the mechanistic bases of these processes [8,9]. Settlement in echinoid species occurs in response to specific settlement cues, typically a chemical released by conspecific adults or from the settlement site itself [10,11]. Historically, much emphasis has been placed on the identification of these species-specific settlement cues. In contrast, developmental mechanisms regulating the acquisition of competence and the physiological, cellular, and molecular processes regulating the metamorphic transition have been understudied [8,9].

*Strongylocentrotus purpuratus* larvae acquire metamorphic competence after 4.5-6 weeks post fertilization depending on the environmental conditions [12]. They settle in response to both red algal turf and crustose coralline algae [13] although the exact chemical identity of the settlement cue remains unknown. In the laboratory, settlement can also be induced using 80 mM excess potassium chloride (KCl) [14]. In *S. purpuratus*, as in most other echinoids with indirect life histories, many juvenile structures are already present within the *S. purpuratus* larva prior to settlement in the form of the juvenile rudiment, a structure required for competency. This is an important adaptation which allows the transition from a metamorphically competent larva to a juvenile to be completed rapidly once the settlement cue is encountered. The major morphological changes that occur during the transition are the resorption of larval tissues, most noticeably the larval arms, and the extrusion and elaboration of the pre-formed juvenile structures, such as the tube feet, which grasp the substrate. In parallel, the larval arms undergo both apoptotic and autophagic cell death [15], and are accompanied by diverse physiological, metabolic and developmental changes (see [16-19] for general description of this process).

Several studies have identified specific neurotransmitters as signalling molecules in cells of the larval nervous system of echinoids, including GABA, serotonin (5HT), SALMFamide-like peptide [20-22], dopamine [23,24], glutamine, and glutamic acid [25]. Some of these compounds, including dopamine, L-DOPA, glutamine and glutamic acid have an inductive role in settlement [24,25]. Recently, Swanson and colleagues identified histamine (HA), another neurotransmitter, as an inducer of settlement in several echinoid species [26-28].

For a signalling molecule to have a modulatory role in metamorphosis, it must a) be synthesized by or is present in specific cells and tissues associated with metamorphic changes and/or the settlement response and b) not have direct inductive functions on competent larvae but alter the rate of settlement upon induction with a specific cue after the larvae have been pre-exposed (for example [29]). While many studies have identified neurotransmitters and peptides in the larval nervous system of echinoids (criteria a), very few studies have performed pharmacological tests to test the involvement of that transmitter in metamorphic competence. Still, based on these criteria, nitric oxide (NO) has been shown to act as a modulator of competence in sea urchins [30-33]. Recent data also suggest that thyroxine may have a modulatory role in settlement [31], in addition to its function as a regulator of larval development [9]. Moreover, as is the case in other cell and tissue level signalling systems, we expect that the physiological and developmental response is the result of interactions between several signalling molecules.

In this study we tested HA as a modulator of metamorphic competence in the larvae of the sea urchin *S. purpuratus*. HA has important functions as both a vertebrate and invertebrate neurotransmitter, and is also produced by bacteria [34,35]. HA is synthesized by histidine decarboxylase (HDC), which is phylogenetically well conserved within bacterial, invertebrate, and vertebrate genomes (see also Additional file 1: Appendix 1 for the relationship of animal sequences). In prokaryotes and eukaryotes, HDC likely evolved independently from a common pyridoxal-dependent decarboxylase [36]. In several arthropod species, HA is the main neurotransmitter of the visual system and evidence suggests that it is also involved in mechanosensory perception, acting through histamine-gated chloride channels [37,38]. In vertebrates, the functions of HA as a neurotransmitter have been studied extensively, and have been linked to a broad range of physiological processes including arousal and alertness, brain metabolism, immune responses, and notably, inhibition of apoptosis [38]. These processes are mediated via one or more of the four known seven transmembrane domain G protein-coupled receptor types, known as H1R through H4R [35]. These receptor types are defined both by their sequence, and their tissue distribution [39,40].

In *S. purpuratus,* HA is an important signalling molecule during fertilization [41]. Antibodies specific to *S. purpuratus* H1R have shown that this receptor is present in the plasma membrane of the egg, and that activation of the receptor by HA sets off a signaling cascade resulting in intracellular calcium release [41]. Here we test whether HA acts as a modulator of metamorphic competence by investigating the effects it has on pre-competent larvae. Furthermore we tested HA involvement as an apoptotic inhibitor of arm retraction in metamorphically competent larvae.

## Materials and methods

### Animals and larval culturing

Adult pacific purple urchins (*S. purpuratus*) were purchased from *The Cultured Abalone Ltd*, Goleta, California (USA), and were housed in an artificial seawater (Instant Ocean™) system in the Hagen Aqualab, University of Guelph, Guelph, Ontario. Adult sea urchins were maintained at a temperature of 12°C and 34‰ salinity under an 8:16 light/dark cycle. The adult urchins were fed rehydrated *Laminaria spp*. (Kombu) kelp once a week (http://www.canadiankelp.com). Gametes were collected by intra-coelomic injection with 0.5-1 mL of 0.5 M KCl or shaking of adult sea urchins. To avoid polyspermy during fertilization a diluted sperm solution was titrated slowly into a dish containing eggs in Millipore™ filtered (0.35 μm) artificial sea water (MFASW). After incubating for 48 h in the fertilization dish at 14°C, cultures were inoculated with approximately 1000 embryos per 1800 mL MFASW in 2 L glass beakers, with numbers subsequently decreasing to 300–500 larvae per culture several weeks later. These beakers were incubated at 14°C under a stir-rack which used paddles placed into each beaker connected to a motor which created a rhythmic disturbance, simulating wave-action and keeping the larvae suspended. The water was changed every two to three days, and at each water change the larvae were fed a combination of both *Dunaliella* and *Isochrisis spp*. (unicellular algae) at a concentration of 6 cells/μl and 12 cells/μl respectively.

### Assay to determine competency and settlement induction

Metamorphic competence cannot be reliably assessed using morphological criteria alone in *S. purpuratus*. Therefore, we measured competence functionally from a random sample that was pooled from all available cultures (larval age between 5–8 weeks). A sub-sample (10–20 larvae) was exposed to either settlement plates grown in artificial seawater or 80 mM excess KCL in MFASW for 1–3 hours to determine the rate of settlement in the cohort. Note that we used the settlement rate resulting from these experiments as an indicator for the percentage of metamorphically competent larvae in that cohort (competence ratio). Settlement plates used for the settlement assay were made by submerging 6 well plastic plates (BD Falcon; 08-772-1B) for 4–6 weeks in tanks filled with recirculating artificial seawater and adult *S. purpuratus*. Depending on the type of experiment planned, we chose a cohort with lower or higher percentages of competent larvae. For example, for pre-incubation with HA we chose a low competency cohort to test pre-treatment with HA, whereas for antagonist and HDC inhibitor (alpha-methylhistidine - AMH) experiments we generally used a cohort of larvae with a high rate of competence (close to 100%) to test for inductive or repressive effects of the compound. In these experiments we tested the hypothesis whether HA acts as an inhibitor of apoptosis and/or settlement once larvae are metamorphically competent.

For all pharmacological tests, three to four replicates of ten larvae were used for each treatment, which included the control, HA (Histamine dichloride - H7250-5 G, Sigma-Aldrich, St. Louis, USA), a HA receptor 1 antagonist (H1Ra; chlorpheniramine - C3025-5 G, Sigma-Aldrich, St. Louis, USA), a HA receptor 2 antagonist (H2Ra; cimetidine - C4522-5 G, Sigma-Aldrich, St. Louis, USA), a HA receptor 3 antagonist (H3Ra, thioperamide - T123-10MG, Sigma-Aldrich, St. Louis, USA), an HDC inhibitor, DL-α-methylhistidine (AMH - SC-285462, Santa Cruz Biotechnology, Santa Cruz, USA), and combinations of HA with antagonists, in 8 mL of MFASW per replicate.

For pharmacological treatments we generally used the minimum exposure time necessary for settlement induction based on preliminary tests. For HA we determined an effective concentration of 1 μM and a minimum pre-exposure time of 24 h hours (we tested 12, 24 and 48 hours - we did observe effects after 12 h as well; not shown). For histamine receptor antagonist experiments we used concentrations previously published for *S. purpuratus *[41] and determined a minimum exposure time of 24 hours. For experiments using AMH, we tested 24, 48 and 72 hours of exposure and found that 48 h was most effective without resulting in toxic effects. The concentrations of AMH tested were 100 μM, 10 μM and 1 μM and we found 100 μM to be most effective which also corresponded with previously published studies [42]. Settlement was tested with either 80 mM excess KCl or settlement plates. Note that results were more consistent with KCl treatment due to variability in settlement plate quality in our artificial seawater system. We also repeated all settlement assays a minimum of 3 times with different cohorts of larvae (different females).

Settlement was scored by a visual inspection of the individual (larva/juvenile) under a dissecting microscope. If the individual had lost the majority of larval characteristics (for example the larval arms had disappeared), had acquired protruding tube feet and spines, and was attached to the substrate, it was scored as settled. If larval characteristics were not detectable, but juvenile characteristics were not apparent (for example the larval arms had disappeared but there were no tube feet or spines present), this was not counted as settlement.

### Immunolocalizations

Embryos and larvae at various stages were fixed for whole mount immunohistochemistry (WMIHC) using 4% EDAC (N-(3-dimethylaminopropyl)-N’-ethylcarbodiimide hydrochloride) in seawater. Note that EDAC is the preferred fixative for HA immunodetection [43]. We also tested EDAC fixation in combination with 4% paraformaldehyde and this resulted in comparable immunodetection. Fixation periods ranged from 20 minutes for embryos and early pluteus larvae to 3 hours at room temperature (RT) for later stages. Specimens were rinsed 3 times in PBST (0.3% TritonX-100 in 1x PBS) for 10 minutes each, incubated in 1% goat serum in PBST, then incubated for 12 hours in HA antibody (Abcam # ab43870-100; Lot 740889) at 1:1000 dilution in 1% goat serum in PBST. Specimens were then rinsed 10 times for 10 minutes each in PBST and transferred to the secondary antibody in PBST (FITC Goat anti-Rabbit IgG 1:400) for 6 hours at RT. After the incubation, specimens were rinsed 10 times for 10 minutes each in PBST and mounted in DABCO (see above). For negative controls, the HA antibody was pre-absorbed with HA-BSA conjugate overnight (>12 hours) and then used as the primary antibody. Note that all WMIHC were performed with the Intavis In Situ Pro liquid handling robot (Intavis, Koeln). Similar protocols were used for the histamine receptor antibody with the following modifications: The histamine receptor antibody was custom made against the sea urchin histamine receptor 1 (suH1R) *in S. purpuratus* (for details see [41]). This antibody was used at the previously determined concentration of 1:500 [41]. In order to assess the effects of HA treatment on receptor endocytosis we exposed eggs to 1uM HA in combination with Texas Red™ (Invitrogen, Grand Island, USA) to mark the lumen of endocytotic vesicles. Eggs were fixed in 4% paraformaldehyde after 10 minutes of incubation and WIHC was performed using suH1R antibody.

### Apoptosis and arm retraction assays

xWe tested for apoptotic activity in competent larvae using the fluorescent-labelled carboxyfluorescein (FAM)-labeled peptide fluoromethyl ketone (FMK) caspase inhibitor (FAM-VAD-FMK) (FAM100-2, Biocarta Cell Technology, San Diego, USA). The FAM-VAD-FMK system has been found to reliably detect apoptotic cells in sea urchin embryos [44] and it can be used to quantify caspase-mediated apoptosis *in situ*. Five-to-ten larvae were pre-incubated in 1 ml of MFASW containing test histamine receptor antagonists, whereas for AMH exposures we used 24 hour incubations based on the results of preliminary experiments, and for the KCl exposure a 0.5 hour exposure time was used. Following this exposure repertoire, 1 μL of FAM-VAD-FMK was added to the dish with larvae, mixed, and incubated for an additional hour. Larvae were then washed five times in 1 mL of MFASW before being fixed with 4% paraformaldehyde (PF) in 1x PBS on ice. After two further washes in 1x PBS, 1 μL of the nuclear fluorescent stain DRAQ5 (Biostatus limited, Shepshed, UK) was added. A 5 minute incubation in the dark was followed by another wash in 1x PBS, after which larvae were mounted in glycerol with 1% DABCO (1,4-diazabicyclo-[2.2.2]octane) pH adjusted to 8–9, in order to extend the life of the fluorescent dyes. Larvae were visualized on a Nikon Eclipse Ti microscope equipped with epiflourescence (521 nm for FAM-VAD-FMK and 670 nm for DRAQ5) using standardized exposure times. To avoid observer bias, larval measurements were randomized by treatment and measurements were independently performed by two observers. Fluorescence intensities were then analyzed in imageJ using standardized area and background. Specifically, the area of the arm to be measured was selected and both the intensity and area were measured resulting in a relative value of intensity per area. The same regions of the larval arms were quantified between treatments and epifluorescence was used to capture total signal within the tissue. Images were standardized by using equal fluorescent exposure and intensity.

**Figure 1:**
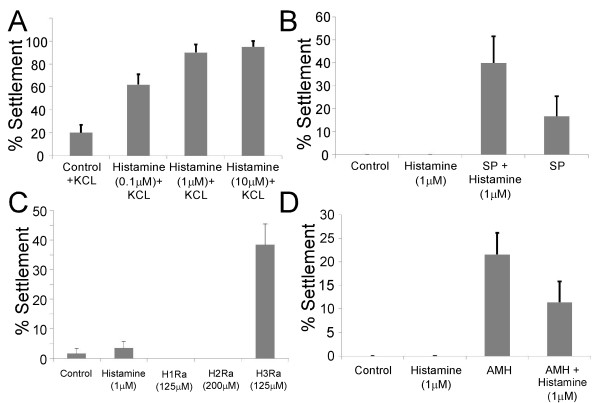
**Pre-treatment of larvae with histamine (HA) leads to an increase of metamorphic competence based on a settlement induction assay with KCl (A) or settlement plates (SP) (B). **Treatment of competent larvae with histamine (HA) receptor 1 antagonist (125 μM chlorpheniramine) and receptor 3 antagonist (125 μM thioperamide) leads to a reduction of arm length measured by the length of the exposed skeletal element from the extent of the arm flesh. HA receptor 2 antagonist (200 μM cimetidine) and HA (1 μM) did not significantly affect relative arm length (A). HA alone did not affect relative arm length in either experiment. Panel B illustrates the measurement of the arm flesh length subtracted from the skeletal element length, giving total exposed skeletal rod length. Note that larval arm length was measured within 24 hours of exposure to treatment. Panel C shows representative larvae that were measured in these experiments. Scale bar: 150 μm.

Pharmacological treatments to measure effect on arm retraction were performed similarly as the settlement assay above. Larvae were exposed to HA receptor antagonists for 24 hours at the same concentrations as in the settlement assay and then were mounted onto slides immediately and photographed. Arm retraction was directly measured from these photographs in imageJ using the straight line feature. To quantify arm retraction, the length of the arm tissue was subtracted from the length of the entire skeletal element, both measurements originating at the same baseline near the stomach or juvenile rudiment, giving the total length of exposed skeletal rod (Figure 1B). In cases with no arm retraction, or where the skeletal element was completely housed by a cellular shroud, the length was calculated as 0.

### Phylogenetic analysis

We identified histamine receptor and HDC sequences from the sea urchin genome (Spbase - http://sugp.caltech.edu/SpBase/). These sequences were aligned against selected vertebrate and invertebrate sequences (for details see Additional file 1: Appendix 1 and Additional file 2: Appendix 2) and analyzed using SeaView (http://pbil.univ-lyon1.fr/software/seaview.html). Specifically we used muscle alignment (default options) and PhyML with LG model, aLRT branch support, no invariable sites, NNI tree searching operations and BioNJ with optimized tree topology.

### Statistical analysis

All data were graphed in Microsoft Excel 2010 and analyzed using SPSS. Standard ANOVA with post-hoc comparisons were used. All data were evaluated for normal distribution using Q-Q and P-P plots. All data used for ANOVA were approximately normally distributed. Note that only absolute values were used for statistical analysis and therefore no additional transformation of data was necessary.

## Results

We tested the effects of histamine signalling on sea urchin metamorphic competence, arm retraction and apoptotic activity. We will use the following abbreviations for treatments throughout this section: histamine receptor 1 antagonist (chlorpheniramine) – **H1Ra**; histamine receptor 2 antagonist (cimetidine) – **H2Ra**; histamine receptor 3 antagonist (Thioperamide) – **H3Ra**; histamine - **HA**; HDC inhibitor (alpha-methylhistidine) – **AMH**. Note that all average values reported here as part of a post-hoc ANOVA are presented as mean differences between treatment groups ± one standard error.

### Pre-treatment of S. purpuratus larvae with histamine leads to attainment of metamorphic competence based on a settlement induction assay

**Figure 2:**
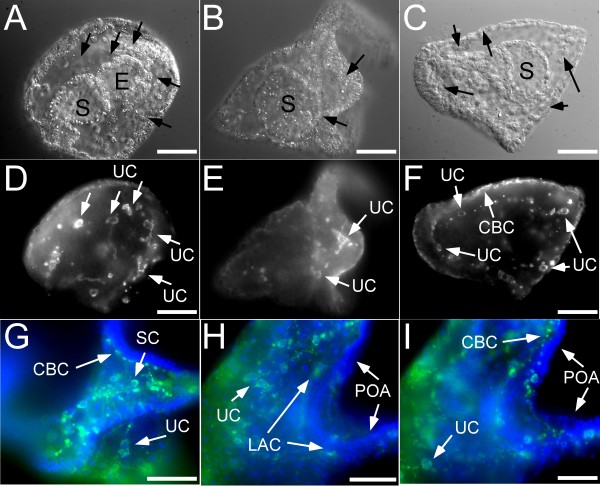
**Pre-treatment of larvae with histamine (HA) leads to an increase of metamorphic competence based on a settlement induction assay with KCl (A) or settlement plates (SP) (B).** Treatment of competent larvae with HA receptor 3 antagonist (C) and HA synthesis inhibitor (D) leads to induction of settlement. A cohort of larvae consisting of both, metamorphically competent and non-competent larvae (20% competence – (see Materials and Methods for details and replicates of experiment) were pre-treated with HA for 24 hours in filtered seawater in the complete absence of food and then exposed to the settlement induction assay. A) Larvae pre-exposed to HA settled at a significantly higher rate when exposed to KCl, a known artificial inducer of metamorphosis in sea urchin larvae compared to controls. (B) Larvae from the HA and control (no HA pre-exposure) did not undergo settlement without induction. After exposure to SP, histamine pre-treated larvae settled at a significantly higher rate compared to control larvae (no HA pre-exposure). We then tested the effect of histamine synthesis inhibitor and several HA receptor antagonists (note that larvae cohorts tested in these experiments were 80-100% competent). (C) Treatment of competent larvae with histamine receptor 3 antagonist (125 μM Thioperamide) for 24 hours leads to a significant increase in settlement without induction by KCl or SP. Histamine receptor 1 (125 μM chlorpheniramine) and receptor 2 (200 μM cimetidine) antagonists did not have any such direct effects on settlement rate. (D) Application of DL-alpha-methylhistidine (AMH, 100 μM), a known histidine decarboxylase (HDC) inhibitor to competent larvae for 72 h also led to a slight increase in settlement rate without induction.

We used the settlement induction assay to test for metamorphic competence in *S. purpuratus* larvae. For these experiments we used cohorts of larvae of the same age with relatively low competence ratios (~20%) in order to better scale an effect. Larvae were pretreated with or without HA, and then induced to settle using either 80 mM KCl or settlement plates. The quality of the settlement plates in the ASW flow-through tanks was highly variable, hence dose response tests (Figure 2A) were performed using 80 mM KCl. Larvae were exposed to different HA concentrations ranging from 0.1 μM to 10 μM for 24 hours and then exposed to 80 mM excess KCl (1–2 hour exposure). Settlement rates for the 1 μM and 10 μM HA pre-treatment were significantly higher than the control (no pre-exposure to HA) (75% ± 11.5; p < 0.01 and 70% ± 11.3; p < 0.01 respectively; Figure 2A). Pre-treatment of 0.1 μM HA did not result in a significant change of the settlement rate compared to the control. From these tests, the optimal concentration of 1 μM HA was determined and used for all subsequent treatments. Figure 2B shows that HA treatment alone did not induce settlement in *S. purpuratus*. If larvae were pre-treated with HA however, they settled at a higher rate upon exposure to settlement plates compared to larvae that were not pre-treated (23.3% ± 10.3, p = 0.05).

### H3Ra and HDC inhibitor (AMH) induce settlement in competent larvae in the absence of cue

We tested the effect of HA, three HA receptor antagonists, as well as an HDC inhibitor on the settlement rate in cohorts of highly (>90%) metamorphically competent larvae (Figure 2C,D). Based on previously published data [39] and our own preliminary assays we used H1Ra and H3Ra at a concentration of 125 μM, and H2Ra at 200 μM (see also Materials and Methods). H3Ra was the only antagonist that significantly increased the percentage of settlement without induction by settlement plates or KCL after 24 hours of exposure (Figure 2C; 36.7% ± 4; F_3_ = 24.2; p < 0.01). In some trials, we also observed a small percentage of settlement without induction in the HA and control treatments (Figure 2C), however settlement rates were very low (<5%; Figure 2C) compared to the nearly 100% rates observed when larvae were induced using KCL in trials prior to the assay (see Materials and Methods for details).

AMH (100 μM), a potent HDC inhibitor [41], increased the rate of settlement without induction relative to controls and HA treatment (Figure 2D; 21.5% ± 5; F_3_ = 7; p < 0.01). This effect was partially reduced when we applied AMH in combination with HA, however the difference between AMH and AMH + HA treatment was not statistically significant (Figure 2D; 10.1% ± 5; F_3_ = 7; p = 0.33).

### Histamine is present in sets of histaminergic cells during early embryonic development and distinct neuronal clusters during metamorphic development

**Figure 3:**
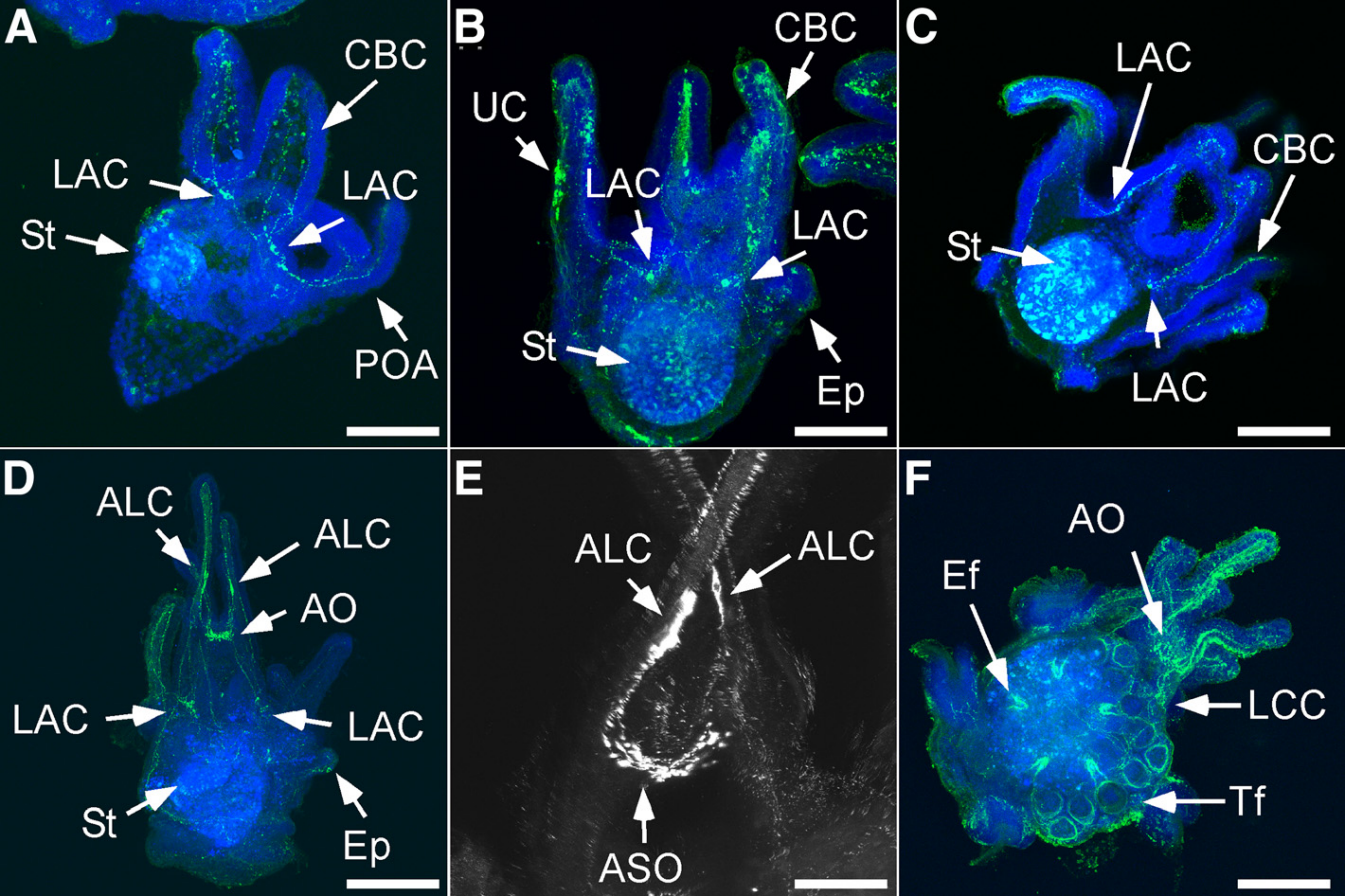
**Histamine (HA) distribution in embryonic stages of the sea urchin*S. purpuratus*.** We used a polyclonal HA antibody for whole mount immunohistochemical (WMIHC) detection of HA in embryonic and post-embryonic (see Figure 4) stages. Panels A-F show late gastrula and early prism stages of the sea urchin *S. pupuratus* visualized using confocal microscopy. A-C are DIC (Differential Interference Contrast) images of panels D-F. Panels G-I are epiflourescent images of late prism to early pluteus stages. We detected two types of histaminergic cells indicated by white arrows in the panels. The first type are putative skeletal cells (SC) the second type are found at the base of the ciliated band (CBC). Note that we also found several unknown cell types (UC) showing HA immunoreactivity. In the early pluteus stages, cells at base of the ciliated band are clearly visible. We were also able to detect similar cells in the region of the aboral lip and in the ciliated epithelium of the larval arms POA – post oral arms). Early pluteus larvae also clearly show the development of the histaminergic lateral arm cluster (LAC). Blue represents a nuclear stain (DRAQ-5) and green indicates detection of HA antibody. Black arrows in DIC images (A-C) indicate corresponding regions of white arrows. Note that DIC images (A-C) are individual cross sections, while corresponding fluorescent images (D-F) are maximum projections. Therefore cells surface regions are not visible in all DIC images. The ubiquitous green stain on the right (BG) illustrates diffuse background fluorescence also seen in control samples (not shown). Scale bars: A,D - 30 μm; B,E - 35 μm; C,F - 25 μm; G,H,I - 20 μm, Abbreviations: S: Stomach; E: Esophagus; UC: Unkown cell types.

We performed whole mount immunohistochemistry (WMIHC) using an antibody to HA on *S. purpuratus* larvae throughout development (Figures 2 and 3). Based on comparisons with pre-absorbed, conjugated antibody, we conclude that although some non-specific binding of the antibody occurs in the stomach and the arms, staining in neurons, ciliated cells, putative pigment (PC) and skeletal cells (SC) further discussed below is specific as it was not detected in the control groups. Non-specific staining is primarily seen in early stages and is generally very diffuse (as seen in Figure 3G-I; see legend for details).

**Figure 4:**
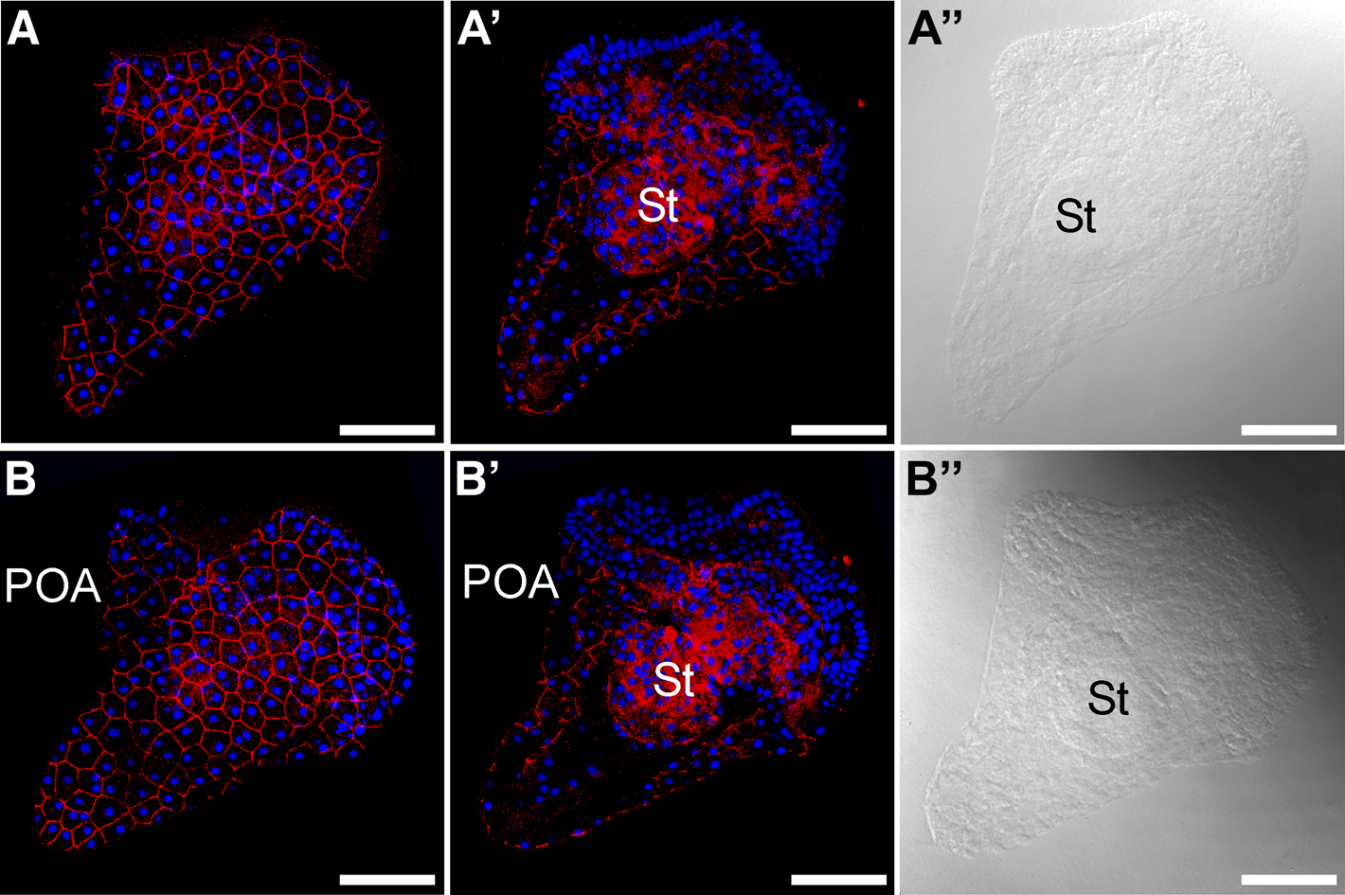
**Histamine (HA) distribution in embryonic and post-embryonic stages of the sea urchin*S. purpuratus*.** Panels A-F show larval stages with increasing age. Nonspecific staining is seen in the stomach (St) in panels A-D. A) 4 arm pluteus larva that shows the developing lateral arm cluster (LAC) at the base of the post-oral arms (POA; indicated by arrows). These cells project into the arms where histaminergic cells are visible in the region of the larval skeletons. HA is also detected in ciliated band cells (CBC) along the larval arms. B) At the 6 arm stage, the lateral cell clusters (LAC) have grown in size and extensive projections into both the anterior and posterior parts of the larva have developed. We also noticed an increase of histaminergic cells in the region of the larval skeletons and the CBC. Unknown cell types (UC) in the leftmost arm and the epaulette (Ep) also show some histaminergic immunoreactivity. Projections into the larval arms can be well seen in panels C and D) At metamorphic competence, a second cluster of histaminergic cells has developed in the region of the apical organ (AO). Extensive projections are now established in all parts of the larva. In addition, two large histaminergic cells were identified in the aboral arms (ALC). These cells are shown at a higher magnification in E. F) shows a different perspective of a competent larva, emphasizing the developing juvenile rudiment. Two main cell types appear to be histaminergic: the epineural folds (Ef) and the epidermis of the tube feet (Tf). The white arrow also depicts the histaminergic cells in the apical organ (AO). Scale bars:A - 75 μm; B - 90 μm; C - 90 μm; F - 150 μm; D - 150 μm; E - 50 μm.

We detected several unknown cell types (UC) in early embryonic stages exhibiting granular HA immunoreactivity (Figure 3). These cells superficially resemble those previously described by Markova et al. [45] based on the interaction of monoamines with glyoxylic acid. These cells have not been further characterized but show superficial similarity to subpopulations of putative sea urchin immune cells [46]. Similar cells have previously been shown to release HA in adult echinoderms [47]. In the prism stage we detected histaminergic cells along the ciliated band (CBC; Figure 3F). In later stages (see below) we detected high levels of HA immunoreactivity in larval skeletal cells based on their proximity to larval skeletal structures (SC Figure 3G-I), specifically in the region adjacent to the skeletal arm rods of the larva.

The first indications of the lateral arm cluster (LAC) became visible in early pluteus larvae with HA immunoreactivity (LAC; Figure 3H). This cluster is also documented in Figure 4 during later developmental stages. No immunoreactivity was detected in the stomach and esophagus region of late gastrula and early pluteus stages (Figure 3). Also, consistent with early stages, we identified staining in cells associated with the ciliated band (CBC; Figures 3, 4A-C). In pre-metamorphic larval stages (2-arm to 8 arm stage), we observed development of the LAC (Figure 4A-C). It appears that cells of this cluster primarily grow in size and not in numbers although we were unable to get an accurate count for the number of cells. Based on our observation of confocal micrographs we estimate 1–4 cells per cluster on each side of the larvae.

The second neuronal cluster is only present at metamorphic competence and consists of cells that are part of the apical organ of the larva (AO). Both clusters send projections into the larval arms and in later stages towards the posterior region of the larva (Figure 4B-F). At metamorphic competence we noted two very distinct cells in the antero-lateral larval arms that have projections to the AO (ALC; Figure 4E).

At metamorphic stages we found a diversity of tissues on the juvenile rudiment that are histamine-positive. These include the epithelial region of the tube feet and the ingressing epineural folds (Figure 4F). We note the identification of a few connections between the AO and the juvenile rudiment (Figure 4F).

### The *S. purpuratus* H1R is expressed throughout development, beginning in the oocyte

**Figure 5:**
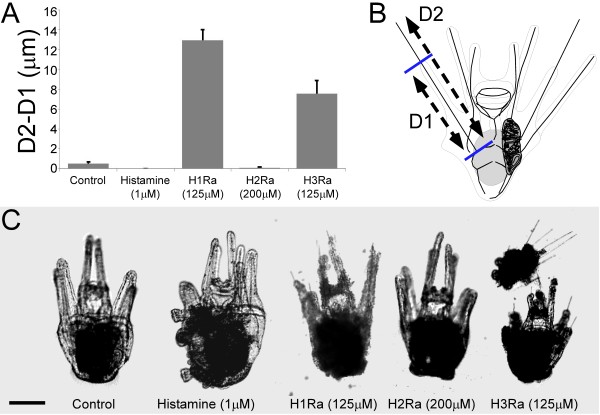
**The sea urchin histamine 1 receptor (suH1R) is localized in the plasma membrane of ectodermal cells in early pluteus stages.** Whole mounted embryos were labeled with anti-suH1R antibodies (in red) and counterstained with Hoechst (in blue) to show nuclei. A and B show confocal images of the surface of the embryo; A’ and B’ show images taken at the equatorial plane; A” and B” show DIC images. St: Stomach; POA: Post-oral arm. Scale bars: 20 μm.

The H1R was visualized in the oocyte (as described in [41]), late blastula, pluteus, and competent larval stages using immunofluorescence with a *S. purpuratus* H1R-specific antibody (Figure 5). H1R is expressed in the plasma membrane of the *S. purpuratus* oocyte, and is internalized into endocytotic vesicles upon HA binding (Figure 5A,B). In larvae of the late blastula stage, H1R expression is predominantly concentrated in the cells of the vegetal plate, while in the early pluteus stage H1R is nearly universally present throughout larval tissues (Figure 5C,D).

### Histamine receptor antagonists and HDC inhibitor lead to a reduction in arm length by caspase mediated apoptosis

**Figure 6:**
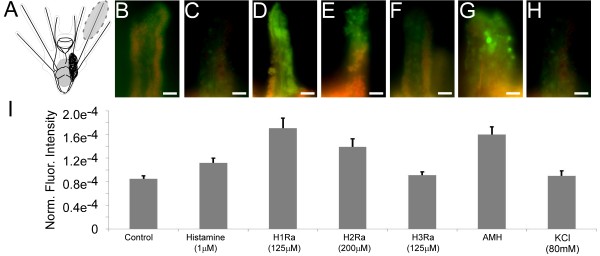
**Histamine (HA) receptor 1 antagonist (125 μM chlorpheniramine) and histidine decarboxylase (HDC) inhibitor alpha-methylhistidine (AMH, 100 μM) treatment of competent larvae leads to increased caspase activity.** Caspase activity was analyzed using FAM-VAD-FMK, a fluorescently tagged caspase inhibitor. Normalized fluorescence was measured in the arm tips of competent larvae. HA, HA receptor 2 antagonist (200 μM cimetidine), HA receptor 3 antagonist (125 μM Thioperamide) and KCl had no effect on caspase activity. The upper panel shows representative fluorescent images of treatment categories: B-control, C-HA (1 μM), D- HA receptor 1 antagonist (125 μM chlorpheniramine), E-HA receptor 2 antagonist (200 μM cimetidine), F-HA receptor 3 antagonist (125 μM Thioperamide), G-alpha-methylhistidine (AMH, 100 μM) and H-KCl. The lower panel shows the corresponding results of the fluorescent analysis. Panel A illustrates the approximate region of the arms that was included in the analysis. Note that all fluorescent intensities were normalized to the area measured and the exposure time. Scale bars: 20 μm.

**Figure 7:**
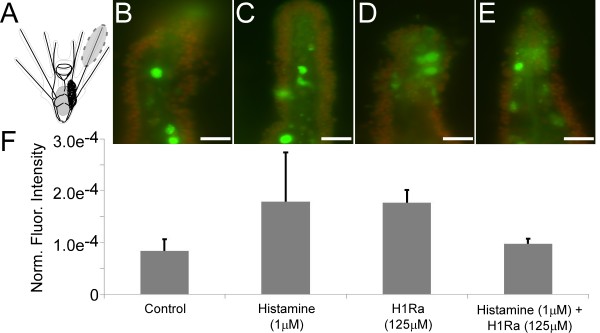
**Histamine receptor 1 antagonist (125 μM chlorpheniramine) did not affect caspase activity in pre-competent larvae.** Caspase activity was analyzed using FAM-VAD-FMK, a fluorescently tagged caspase inhibitor. Overall fluorescence was measured in the arms tips of competent larvae. Histamine (1 μM), Histamine receptor 1 antagonist (125 μM chlorpheniramine) and histamine (1 μM) plus Histamine receptor 1 antagonist (125 μM chlorpheniramine) treatment had no significant effect on caspase activity. Scale bars: 20 μm.

Metamorphically competent larvae retract their arms in response to a settlement cue such as KCL or settlement plates, as a part of metamorphosis. We tested the effects of HA, HA receptor antagonists and AMH on arm retraction and caspase mediated apoptosis (Figures 1, 6, 7). Treatment of larvae with H1Ra and H3Ra (both at 125 μM) resulted in significant arm retraction after 24 hours compared to the control, and quantitated by the length of the exposed arm skeletal element (Figure 1A,B; 12.43 ± 0.89 μm and 7.04 ± 1.16 μm respectively; F_4_ = 68.77, p < 0.001). H1Ra had a significantly greater effect compared to H3Ra (5.39 ± 0.26 μm longer, p < 0.001), however it should be noted that several individuals treated with H3Ra had settled spontaneously by the time of measurement, leaving no larval morphology intact, making it impossible to determine the extent of arm retraction. The arm retraction results include only those individuals which still had larval structures intact after the 24 hour treatment which could be measured. H2Ra (200 μM) and HA (1 μM) did not have a significant effect on arm retraction. The pattern of arm retraction in response to H1Ra and H3Ra was very different (Figure 1C). Arm retraction in response to H3Ra was very similar to retraction seen in a settling individual, while H1Ra treatment led to what appeared to be random cell death in a response independent of metamorphic processes (Figure 1C). Larvae treated with HA or H2Ra are both comparable to controls after 24 hours (Figure 1C).

In metamorphically competent larvae, caspase-dependant fluorescence based on fluorescently labelled caspase inhibitor (FAM-VAD-FMK) activity in the arm tips was significantly higher when larvae were exposed to 125 μM H1Ra (Figure 6; 6 × 10^-5^ ± 9 × 10^-6^; F_6_ = 15.3; p < 0.01), 200 μM H2Ra (Figure 6; 4 × 10^-5^ ± 1 × 10^-5^; F_6_ = 15.3; p < 0.01) and 100 μM AMH (Figure 6; 6 × 10^-5^ ± 1 × 10^-5^; F_6_ = 15.3; p < 0.01) compared to controls. In contrast, H3Ra treatment at 125 μM had no significant effect on caspase activity compared to controls (Figure 6; 1x10^-5^ ± 9 × 10^-6^; F_6_ = 15.3; p = 1). We also found no effect of 1 μM HA (Figure 6; 1 × 10^-5^ ± 9 × 10^-6^; F_6_ = 15.3; p = 1), nor a 0.5 hour treatment with 80 mM excess KCl (Figure 6; 1 × 10^-6^ ± 1 × 10^-5^; F_6_ = 15.3; p = 1) on caspase activity. This experiment was not performed using settlement plates due to the complications which arise with its variable quality.

In contrast to these effects on competent larvae, we did not detect any effect of H1Ra (125 μM) on caspase activity in pre-competent larvae (Figure 7; 8 × 10^-5^ ± 6 × 10^-5^; F_3_ = 1.32; p = 1). Note however that a combined treatment of HA with H1Ra resulted in a slight but non-significant reduction of caspase activity in comparison to H1Ra treatment alone (Figure 7; 8 × 10^-5^ ± 6 × 10^-5^; F_3_ = 1.32; p = 0.16). Due to these results, we did not test AMH or any other HA antagonist on pre-competent stages. Table 1 provides a summary of all HA related pharmacological effects on settlement, arm retraction and caspase activity for competent larvae.

**Table 1:** Summary of the effects of HDC inhibitor, HA, HA receptor antagonists and KCl on settlement without induction, arm retraction and caspase activity for competent larvae

**Treatment**	**Settlement**	**Rel. Arm Length**	**Caspase Activity**
HA	**-**	**-**	**-**
AMH	**↑**	**-**	**↑**
H1Ra	**-**	**↓**	**↑**
H2Ra	**-**	**-**	**↑**
H3Ra	↑	**↓**	**-**
KCL	↑	**↓**	**-**

All effects indicated in this table were statistically significant (p < 0.05).

all results significant for p < 0.05.

## Discussion

Many laboratory and field studies support the idea that both stimulatory and inhibitory factors influence the extent of the competent stage and that food concentration, endogenous resources and timing within the life cycle play an important role in this process (for examples [48-57]). Our data provide evidence for histamine (HA) function in metamorphic competence. Specifically, we show that; 1) HA acts as a developmental signal leading to pre-competent larvae acquiring competence; 2) HA is concentrated in nervous centers implicated in the settlement response in other species; 3) inhibition of H3R or HA synthesis results in spontaneous settlement; and 4) HA has inhibitory effects on caspase mediated arm retraction in metamorphically competent larvae.

### Histamine leads to the acquisition of metamorphic competence in *S. purpuratus*

It has been suggested that neurons of the AO are involved in detecting environmental information (i.e. settlement cues) and translate that information into physiological and behavioural responses during the metamorphic transition [24,29,58-60]. Confocal analysis of the histaminergic cluster in the AO reveals a complex network of neuronal connections to anterior and posterior targets including neurons in the anterolateral larval arms. While the function of these neurons remains to be elucidated, the fact that they are associated with the AO indicates that there may be a link between HA signalling and other neuronal signalling systems in the larvae (see also below for further discussion).

**Figure 8:**
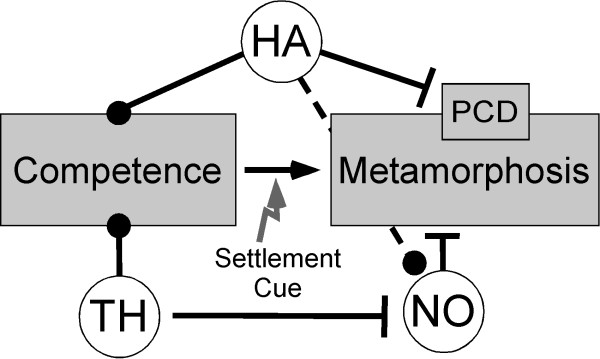
**Proposed model of modulatory actions in metamorphic competence and settlement of sea urchin larvae.** Round symbols indicate agonistic relationships while flat lines indicate antagonistic interactions. Data presented here suggests a modulatory role of histamine (HA) in metamorphic competence. Based on our data we propose that HA leads to the acquisition of competence in pre-competent larvae and maintains competence in competent larvae by inhibiting caspase mediated apoptosis. Previously published data also suggest a function of thyroid hormones and nitric oxide (NO) signaling in modulating metamorphosis. Note that published data [40] established a link between NO and HA signaling in the sea urchin *S. purpuratus*. Future research will focus on the interactions of these signaling pathways in metamorphic competence and settlement

We hypothesize a dual function of histamine in metamorphic development: 1) it functions on pre-competent larvae to become competent and 2) once larvae are metamorphically competent, HA maintains that developmental priming by inhibiting apoptosis and settlement in the absence of a settlement cue. We have three lines of evidence supporting this hypothesis: 1) AMH treatment of competent larvae leads to a higher settlement rate compared to the control; 2) HR3 antagonist treatment leads to a higher settlement rate compared to the control; and 3) HA treatment of competent larvae does not lead to an increase of caspase mediated apoptosis. Nitric oxide (NO) has also been shown to function as an inhibitory signal for settlement in competent larvae. Evidence for such a role has been documented for sea urchins [30,32,58,61], ascidians [58] and mollusks [62]. We also showed that another metamorphic signal, thyroxine [63], is correlated with a reduction in the number of neurons containing nitric oxide synthetase (NOS) in metamorphically competent larvae of the sea urchin *Lytechnius pictus *[31], and through diminishing the expression of this repressive signal, it may act as a metamorphic inducer. Leguia and Wessel [41] documented that HA leads to the activation of NO signalling during sea urchin fertilization. Therefore, we predict that HA may act agonistically through NO signaling during metamorphic competence (Figure 8). At this point it is unclear why H3R antagonist treatment of competent larvae was the only antagonist treatment to lead to a settlement response. However, it is important to note that the only functionally characterized histamine receptor is H1R (suH1R – [41]) and we do not know the specificity of antagonist treatments on any of the receptors present in the sea urchin. Sequences of the histamine receptor family members in *S. purpuratus* though are very similar to their vertebrate orthologs [41]. Future work will focus on identifying the other two sea urchin histamine receptors (see also below further discussion of putative HA receptors in the sea urchin). Such experiments will also help to further evaluate a potential link between NO and HA signalling in metamorphic competence.

Settlement strategies of marine invertebrate larvae range from very opportunistic strategies, where competent larvae adjust their specific settlement requirements as the competent stage advances, to strategies where such adjustments rarely occur, and larvae can spend considerable time in the plankton until suitable settlement sites are found [5,31,50]. For example, *S. purpuratus* larvae generally show a relatively low tendency to settle in the absence of specific cues (our own unpublished data and data presented here). In contrast, certain clypeasteroid species, such as *Clypeaster rosaceus* frequently settle under experimental conditions in the complete absence of specific settlement cues [64]. We have previously argued that the mechanisms underlying opportunistic settlement strategies likely involve neuro-modulatory signalling systems, which integrate developmental, nutritional, energetic, as well as multiple sensory inputs integrated within different signalling pathways [8]. Evolutionary changes in settlement strategies within and among marine invertebrate taxa likely involve changes in these modulatory systems. Future research should therefore test whether differences in HA signalling exist between closely related echinoid species with different settlement strategies to begin to test how changes in life history might evolve.

To this end, a comparison between *H. purpurascens* and *S. purpuratus* indicates that important differences in HA signalling do exist. Previous work by Swanson et al. [26-28] has shown that exposure of some echinoid species to HA induces settlement but they did not find any clear pattern with respect to developmental mode or phylogeny. These authors also identified HA in the seawater surrounding algae in the field and provided evidence that HA is released by the algal species that is preferentially settled by larvae. In contrast to these studies on *H. purpurascens*, we did not find any evidence for HA as a settlement inducer in competent *S. purpuratus* larvae. Instead, our data strongly suggest a modulatory function of HA in metamorphic competence.

Despite the different roles of HA in these two species, it could be performing the same signaling function in both, with the difference being in the source of HA. *H. purpurascens* larvae inhabit an environment with algae species known to release HA into the water, and larvae could potentially also be exposed to histamine through their diet, while it is unknown if algae species associated with *S. purpuratus* do the same. Differences in the capacity of algae in the larvae’s respective environments to synthesize HA could affect the use of this signalling system in metamorphic competence. Finally, bacterial communities are an important component of marine biofilms settled by some echinoid species. It will be important to analyze the concentration of HA in the seawater around such biofilms as well as in algal turf and crustose coralline algae to test their role in the settlement response of sea urchin larvae. This is particularly relevant as HA signaling may be involved in the settlement processes of barnacles, bryozoans and polychaetes as well [65]. Specifically, experiments have shown that a H1R antagonist, mizolastine, has inhibitory effects on the settlement response of selected species from these groups [65]. Although no further investigations into the signaling mechanisms of HA have been accomplished, these data suggest that HA signaling may be widespread in the marine environment.

### Histamine is present in neuronal clusters, some with sensory functions in the detection of settlement cues

The development of the embryonic and larval nervous system in *S. purpuratus* has been well studied and several identifiable clusters have been described [66-71]. Neuroblasts begin to form in the late gastrula stage and are closely associated with the developing ciliary band. At later larval stages and metamorphic competence, a complex network of neurons with distinct ganglia is present. These ganglia are the AO, in the anterior region of the larva in between the pre-oral arms, the lateral ganglia, and consist of two clusters at the base of the post-oral arms, and the oral ganglion around the mouth.

One histaminergic cell type that we detected as early as the late gastrula stage resembles the putative echinoderm immune cells (blastocoelar cells). These are known to house HA in adult echinoderms, as well as a red pigment believed to also be involved in the immune response [46,47,72]. We also detected immunoreactive cells associated with the ciliated band, which become more numerous during the prism stage and were found throughout the epithelium along the ciliated band. In later stages these cells appeared closely associated with axonal tracts along the mature ciliated band.

Later in larval development, HA-positive cells appeared in proximity with other components of the larval nervous system as has been previously shown using the pan-neuronal antibody 1E11 [71,73,74]. For example, HA has been implicated as a neurotransmitter in photoreceptive functions [37] and it would be important to further investigate such a role in sea urchin larvae. Recent work has also identified the tube feet as a region where phototransduction occurs [75]. Our data show that HA is expressed in juvenile tube feet, and suggests that HA may function in phototransduction, possibly in association with mechanisms underlying settlement. For example, serotonin and dopamine both effect phototaxis in bryozoans [29] and changes in phototaxis have been described for competent larvae (i.e. [76,77]).

We identified HA expression in cells of the LAC and AO. Histochemical analysis indicates that the appearance of histaminergic neurons in the AO occurs later in development and reaches its maximal extent during metamorphic competence. These data support our conclusion of the proposed role of HA as a modulator of metamorphic competence. Based on our IHC analysis a significant amount of HA is contained and released by neurons of the LAC and AO. The AO of *S. purpuratus* larvae (as for many other marine invertebrate species) is a neuronal structure that is distinct from the adult nervous system and degenerates during settlement [73]. Functions of this organ among echinoids have been linked to settlement and specifically to the detection and modulation of settlement cues [60]. Histaminergic cells in the AO have never been described before and our data indicate a novel function of this transmitter in settlement.

### Histamine inhibits caspase mediated apoptosis in metamorphically competent larvae

Resorption of larval structures during metamorphosis is an important process contributing to overall tissue reorganization not only in echinoderms, but also in amphibians and arthropods [9,78]. Sato et al [15] demonstrated that larvae of another sea urchin species, *H. pulcherrimus*, displayed TUNEL-positive (terminal deoxynucleotidyl transferase (TdT)-mediated biotinylated deoxyuridine-triphosphate (dUTP)-biotin nick-end label) and monodansyl cadaverine (MDC) staining, characteristic of apoptotic and autophagic cell death respectively, in the larval arms during arm retraction when they are induced to settle by a natural settlement inducer. While it was outside the scope of this study to test the specific mechanism of programmed cell death (PCD) in *S. purpuratus* larvae, we used a generic fluorescent-labelled caspase inhibitor (FAM-VAD-FMK) to test whether caspase mediated apoptosis is involved in arm retraction and whether this process is regulated by HA in competent larvae. This approach was also partially motivated by the fact that HA has been previously identified as a regulator of caspase mediated apoptosis [79].

Our data provides preliminary evidence for HA function in inhibiting caspase activity in competent larvae and that this inhibition is mediated by H1R and/or H2R. However, treatment of larvae with antagonists for these receptors did not result in the induction of settlement and only H1R antagonist treatment resulted in arm retraction. In contrast, H3R antagonist treatment resulted in both arm retraction and settlement, but not in increased caspase activity. Even KCl, a reliable, artificial inducer of settlement in many species of sea urchins, did not result in increased caspase activity. Together these results show that although experimental inhibition of certain HA receptors in competent larvae leads to settlement, arm retraction, and increased caspase activity, caspase mediated apoptosis does not appear to be necessary for arm retraction. Furthermore, our results suggest that arm retraction can be decoupled from other metamorphic processes in sea urchin settlement and can be regulated through both caspase dependent and independent mechanisms (for more information on caspase independent apoptosis see [80]). These data are also consistent with work on the mollusk, *Crepidula fornicate* where excess K^+^ appears to induce settlement but via a different pathway then natural inducers [81]. Additional preliminary data from our lab show that competent sea urchin larvae that were treated with a general caspase inhibitor (FAM-VAD-FMK) for several hours retain the capacity to settle. These results further confirm that caspase activity is not a requirement for arm retraction and settlement. Therefore, future experiments will have to test the cellular and molecular processes involved in arm retraction and how they are linked to HA signalling.

HA signal transduction can occur through the binding of the ligand to different types of HA receptors. In mammals, four receptors have been described and these receptors are generally characterized by overlapping tissue specificities [39]. We performed a complete analysis of all HA receptor predictions from the *S. purpuratus* genome (SpBase) and identified three orthologous receptor genes in the sea urchin (Additional file 2: Appendix 2) [73,82]. Based on pairwise sequence alignments and phylogenetic analysis (Additional file 2: Appendix 2), sea urchin receptor 1 and 2 can be clearly assigned to their mammalian counterpart. Still, a preliminary pharmacological screen for HA receptors in settlement and apoptosis revealed diverging functions of different receptor types (see above). In order to gain a more detailed insight into HA receptor functions it will be essential to use additional receptor antagonists and also explore functional genomics approaches using the sea urchin receptor.

## Conclusions

This study has provided evidence for a modulatory function of HA in settlement of *S. purpuratus*. Firstly, HA was demonstrated to lead to the acquisition of metamorphic competence in this species. Secondly, HA was found in several important neuronal clusters, some of which, such as the apical organ (AO), have been previously implicated in mediating settlement responses. Third, caspase-mediated apoptosis is inhibited by HA through both H1R and H2R in competent larvae.

## Abbreviations

HA, Histamine; H(1,2,3,4)R, Histamine (1,2,3,4) receptor; H(1,2,3)Ra, Histamine receptor antagonist; HDC, Histidine Decarboxylase; AMH, alpha-methylhistidine (HDC inhibitor); FAM-VAD-FMK, Carboxyfluorescein (FAM)-labeled peptide fluoromethyl ketone (FMK) caspase inhibitors; PCD, Programmed cell death; AO, Apical organ; ALC, Antero-lateral cells; LAC, Lateral arm cluster; CBC, Ciliated band cells; PC, Putative pigment cells; SC, Skeletal cells; UC, Unknown cells.

## Authors’ contributions

JS performed the settlement assays, arm retraction assays and apoptosis assays. J-LG performed the immunolocalization of the HAergic system in developing larvae. JN provided technical assistance. GW and ML developed the *S. purpuratus* H1R-specific antibody and examined its expression. GW also provided technical assistance and advice on the manuscript. AH drafted the manuscript, designed, and funded all experiments performed by JS, J-LG, and JN. Cultures were maintained by JS, J-LG, JN and AH. All authors read and approved the final manuscript.

## Supplementary Material

Additional file 1 **Appendix 1** We identified and cloned the full length cDNA from the sea urchin histidine decarboxylase (HDC) gene based on the annotation present in NCBI (XP_789367). We then used the protein sequence for phylogenetic analysis and comparison with other animal HDCs as well as three dopa decarboxylase genes (Ddc). HDC from S. purpuratus clearly falls within the HDC cluster of other bilaterian animals and is distinct from the Ddc clade. Click here for file

Additional file 2 **Appendix 2** Neighbor joining phylogenetic analysis of sea urchin histamine receptor predictions in comparison to human H1, H2, H3 and H4 receptor. We identified all histamine-receptor like genes from SpBase (http://sugp.caltech.edu/SpBase/) and aligned them with human H1, H2 and H3 receptor. Phylogenetic analysis suggests that all three receptor types are present in the sea urchin genome. Click here for file
