# Correction: Histamine is a modulator of metamorphic competence in Strongylocentrotus purpuratus (Echinodermata: Echinoidea)

**DOI:** 10.1186/1471-213X-13-30

**Published:** 2013-07-23

**Authors:** Josh Sutherby, Jamie-Lee Giardini, Julia Nguyen, Gary Wessel, Mariana Leguia, Andreas Heyland

**Affiliations:** 1 University of Guelph, Integrative Biology, Guelph ON N1G-2 W1, Canada; 2 Brown University, MCB, Providence RI 02912, USA; 3 Current address: U.S. Naval Medical Research Unit No.6, Lima, Peru

## 

In Figure six (Figure 1 here) of the original manuscript [1] panels B-H are representative images from which measurements were taken and then graphed in Figure six panel I. In the original submission of the manuscript panel C and H ended up showing identical images. We corrected this error by replacing panel C with the correct representative image. Note that this error occurred when preparing the original figure and it does not affect the data presented in any way.

**Figure 1:**
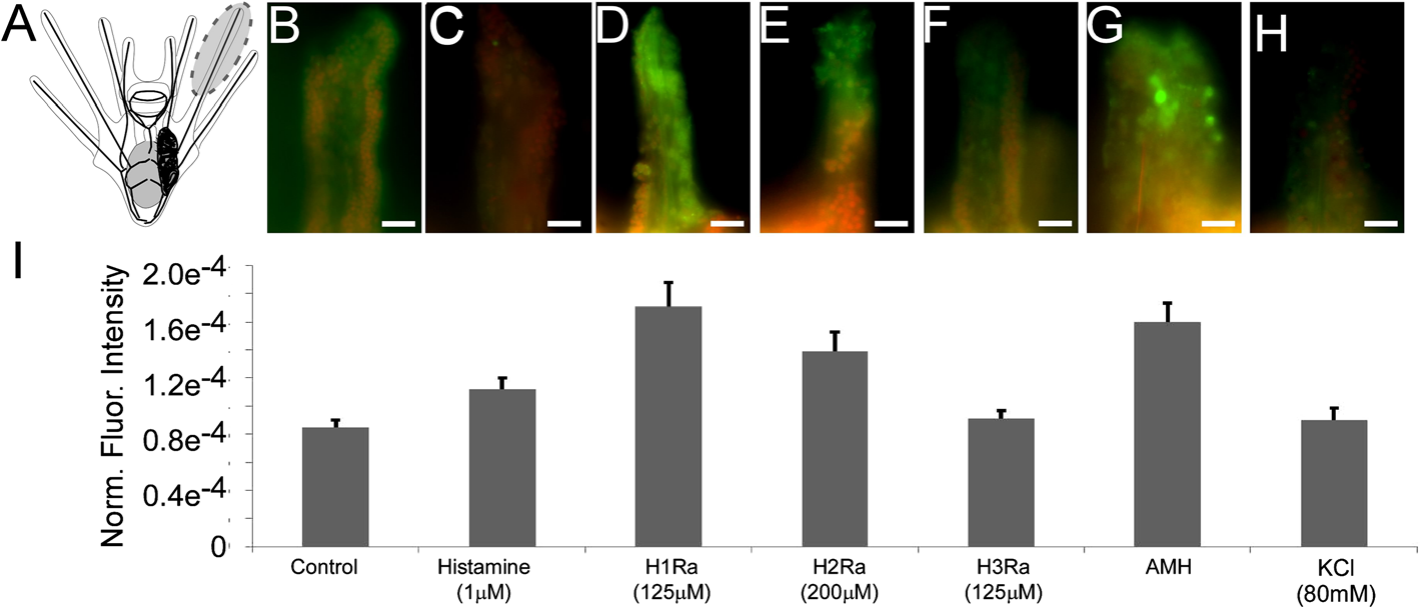
**Histamine (HA) receptor 1 antagonist (125 μM chlorpheniramine) and histidine decarboxylase (HDC) inhibitor alpha-methylhistidine (AMH, 100 μM) treatment of competent larvae leads to increased caspase activity.** Caspase activity was analyzed using FAM-VAD-FMK, a fluorescently tagged caspase inhibitor. Normalized fluorescence was measured in the arm tips of competent larvae. HA, HA receptor 2 antagonist (200 μM cimetidine), HA receptor 3 antagonist (125 μM Thioperamide) and KCl had no effect on caspase activity. The upper panel shows representative fluorescent images of treatment categories: B-control, C-HA (1 μM), D- HA receptor 1 antagonist (125 μM chlorpheniramine), E-HA receptor 2 antagonist (200 μM cimetidine), F-HA receptor 3 antagonist (125 μM Thioperamide), G-alpha-methylhistidine (AMH, 100 μM) and H-KCl. The lower panel shows the corresponding results of the fluorescent analysis. Panel A illustrates the approximate region of the arms that was included in the analysis. Note that all fluorescent intensities were normalized to the area measured and the exposure time. Scale bars: 20 μm.
