# Supplementary figures and images for: Histamine is a modulator of metamorphic competence in Strongylocentrotus purpuratus (Echinodermata: Echinoidea)
Source: BMC Dev Biol. 2012 Apr 27;12:14. doi: 10.1186/1471-213X-12-14 (PMC3460732; doi:10.1186/1471-213X-12-14)

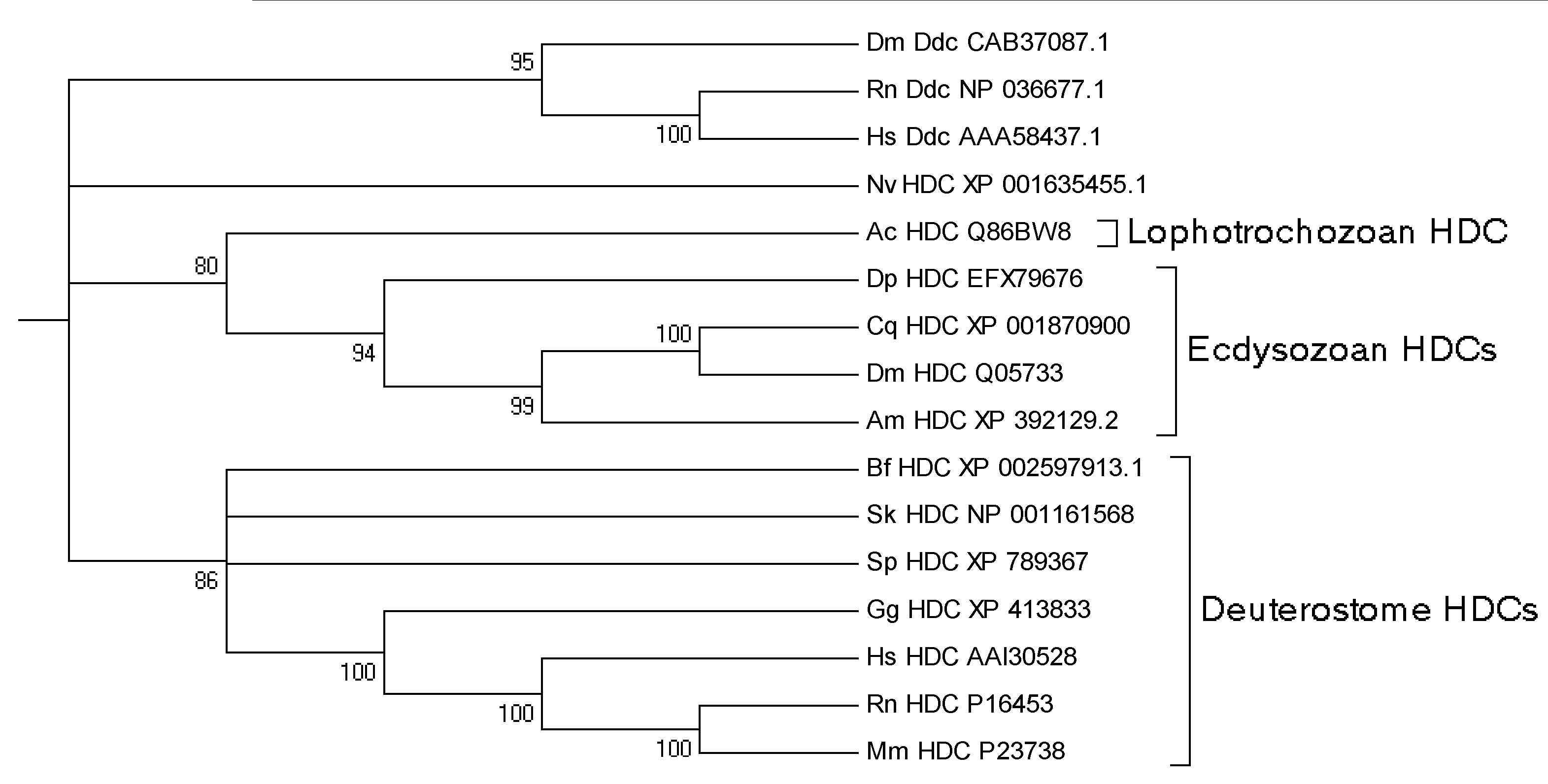

Supplement: Additional file 1 — Appendix 1 We identified and cloned the full length cDNA from the sea urchin histidine decarboxylase (HDC) gene based on the annotation present in NCBI (XP_789367). We then used the protein sequence for phylogenetic analysis and comparison with other animal HDCs as well as three dopa decarboxylase genes (Ddc). HDC from S. purpuratus clearly falls within the HDC cluster of other bilaterian animals and is distinct from the Ddc clade. [file 1471-213X-12-14-S1.jpeg]

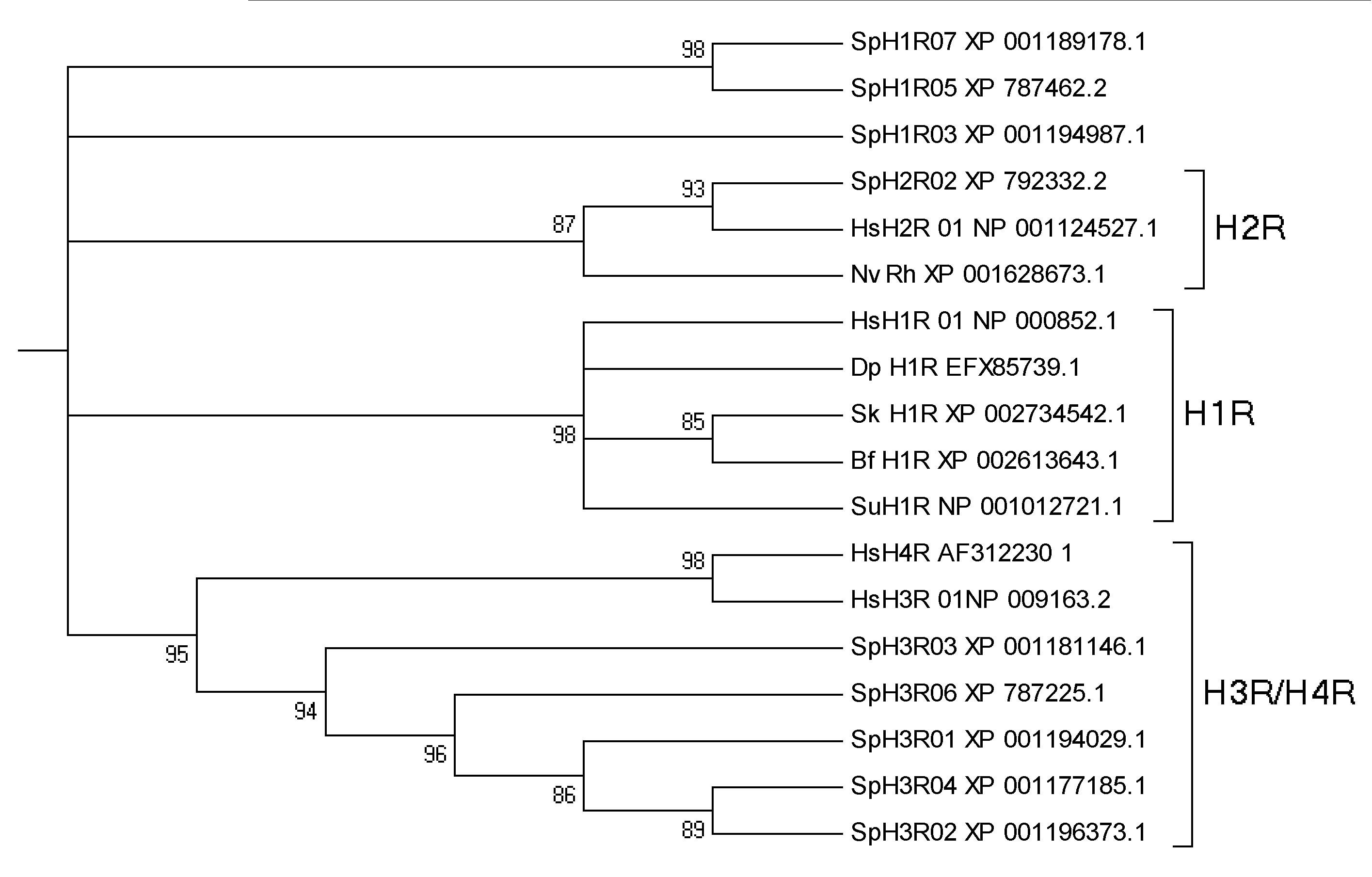

Supplement: Additional file 2 — Appendix 2 Neighbor joining phylogenetic analysis of sea urchin histamine receptor predictions in comparison to human H1, H2, H3 and H4 receptor. We identified all histamine-receptor like genes from SpBase (http://sugp.caltech.edu/SpBase/) and aligned them with human H1, H2 and H3 receptor. Phylogenetic analysis suggests that all three receptor types are present in the sea urchin genome. [file 1471-213X-12-14-S2.jpeg]
